# The cation diffusion facilitator protein MamM's cytoplasmic domain exhibits metal-type dependent binding modes and discriminates against Mn^2+^

**DOI:** 10.1074/jbc.RA120.014145

**Published:** 2021-01-13

**Authors:** Shiran Barber-Zucker, Jenny Hall, Afonso Froes, Sofiya Kolusheva, Fraser MacMillan, Raz Zarivach

**Affiliations:** 1 Department of Life Sciences, Ben-Gurion University of the Negev, Beer Sheva, Israel; 2 The National Institute for Biotechnology in the Negev, Ben-Gurion University of the Negev, Beer Sheva, Israel; 3 Ilse Katz Institute for Nanoscale Science and Technology, Ben-Gurion University of the Negev, Beer Sheva, Israel; 4 Henry Wellcome Unit for Biological EPR, School of Chemistry, University of East Anglia, Norwich, United Kingdom

**Keywords:** structure-function, metalloprotein, metal ion-protein interaction, electron paramagnetic resonance (EPR), X-ray crystallography, cation diffusion facilitator, magnetotactic bacteria, metal selectivity

## Abstract

Cation diffusion facilitator (CDF) proteins are a conserved family of divalent transition metal cation transporters. CDF proteins are usually composed of two domains: the transmembrane domain, in which the metal cations are transported through, and a regulatory cytoplasmic C-terminal domain (CTD). Each CDF protein transports either one specific metal or multiple metals from the cytoplasm, and it is not known whether the CTD takes an active regulatory role in metal recognition and discrimination during cation transport. Here, the model CDF protein MamM, an iron transporter from magnetotactic bacteria, was used to probe the role of the CTD in metal recognition and selectivity. Using a combination of biophysical and structural approaches, the binding of different metals to MamM CTD was characterized. Results reveal that different metals bind distinctively to MamM CTD in terms of their binding sites, thermodynamics, and binding-dependent conformations, both in crystal form and in solution, which suggests a varying level of functional discrimination between CDF domains. Furthermore, these results provide the first direct evidence that CDF CTDs play a role in metal selectivity. We demonstrate that MamM's CTD can discriminate against Mn^2+^, supporting its postulated role in preventing magnetite formation poisoning in magnetotactic bacteria via Mn^2+^ incorporation.

Cation diffusion facilitator (CDF) proteins are a conserved family of divalent transition metal cation transporters. CDF proteins form dimers, commonly homodimers, usually containing two domains: the N-terminal transmembrane domain (TMD) through which cations are transported and the frequently found regulatory cytoplasmic C-terminal domain (CTD) (1, 2, 3, 4, 5). Each CDF protein transports specific metal cations (Zn^2+^, Mn^2+^, Cu^2+^, Co^2+^, Ni^2+^, Cd^2+^, or Fe^2+^) from the cytoplasm to either the extracellular environment or inner-cellular compartments depending on its membranal location, typically by exploiting the proton-motive-force or by utilizing other cation gradients (6, 7, 8, 9, 10). Because they control metal cation homeostasis at the cellular level, CDF proteins are crucial for proper cell function. As an example, malfunction of human CDF proteins leads to severe diseases including cardiovascular diseases, type-II diabetes, parkinsonism, and Alzheimer's disease (11, 12). Although a greater understanding of CDF protein function at the molecular level has been achieved in recent years, the CDF transport mechanism is not yet fully understood. Furthermore, factors governing metal selectivity of these proteins are still under investigation (7); hence the exact structure-function relationship of CDF proteins remains unresolved, specifically how such relationships affect metal selectivity.

Magnetotactic bacteria (MTB) are a group of gram-negative bacteria that align with and move along magnetic fields. The bacterium contains subcellular organelles named magnetosomes, each of which is composed of ∼30–120-nm iron-based magnetic particles (magnetite or greigite) enclosed in a protein-rich lipid membrane. The magnetosome is able to discriminate against different cations, resulting in pure magnetite or greigite particles (13, 14, 15). The magnetosomes are arranged on an actin-like polymer in a chain arrangement, creating a magnetic dipole moment allowing these bacteria to make use of external magnetic fields. In nature, this property enables the bacteria to orient themselves to the geomagnetic field, thus navigating to their preferred habitat, usually oxic-anoxic zones in aquatic environments (16, 17). MamM, a CDF protein found uniquely on the magnetosome membrane, transports iron from the cytoplasm into the magnetosomes, enabling the synthesis of pure magnetic particles. It has been demonstrated that the deletion of the *mamM* gene, or even of its CTD, abolishes magnetic particle formation, emphasizing its importance for the biomineralization process (18). In previous studies, WT MamM CTD was shown to bind iron, and the crystal structure of the apo form adopts the characteristic fold of CDF protein CTDs, the presence of which is crucial for the overall protein function. Additionally, three putative MamM CTD metal-binding sites were proposed that included two symmetrical, allosteric peripheral sites (PSs, composed of His-264 from one monomer and Glu-289 from the second monomer) and one central site (CS, composed of Asp-249 and His-285 from both monomers). Biophysical evidence revealed that zinc binding to the PSs leads to a change in protein conformation from an open, dynamic V-shaped dimer to a much more rigid, tighter dimeric packing (5, 19, 20, 21). These studies and those of other bacterial CDF proteins suggest the following common mechanism for CDF proteins: at a certain cytoplasmic metal cation concentration, cations bind to the CTD, locking it in this tighter packed state as compared with its apo open and dynamic V-shape. This, in turn, facilitates a conformational change of the TMD, thus enabling cations to be transported through this domain to the other side of the membrane (4, 5, 6, 21, 22, 23). Although previous MamM studies characterized some of the roles and the mechanism of the CTD in CDF proteins, one question has remained unanswered: whether the CTD has an active regulatory role in metal recognition and discrimination during cation transport.

In this study, the well-characterized MamM CTD is used as a model to investigate the role of CTDs in metal recognition and selectivity *in vitro*, and this work presents, for the first time, clear structural and spectroscopic evidence of metal binding of any CDF CTD to multiple metals. The crystal structure of MamM CTD was solved in the presence of Cu^2+^, Cd^2+^, and Ni^2+^, revealing the propensity of difference metals to bind at different metal-specific sites, leading to different conformations in the crystalline state. To further characterize the binding abilities of additional metal cations to MamM CTD in solution, tryptophan-fluorescence spectrometry, isothermal titration calorimetry (ITC), and pulsed electron-electron double resonance (PELDOR) spectroscopy were employed. Results indicate that all these metal cations have similar binding abilities to MamM CTD, with the exception of Mn^2+^. Their binding typically leads to the same or a similar tighter, more rigid conformation as that detected for the Cu^2+^-bound crystallographic conformation. Each bound metal, however, does exhibit slightly different thermodynamic binding parameters, which could imply a degree of selectivity for the effectivity of metal binding to the CTD. Overall, the results presented here demonstrate that MamM CTD can recognize different metals and discriminates against Mn^2+^, suggesting an inherent mechanism to prevent its transport by hindering the CDF transport activation, presumably to avoid impure iron-based magnetic particles synthesis. Thus, we see here the first direct evidence for the role of the CTD in metal selectivity in CDF proteins.

## Results and Discussion

### Crystal structures of MamM CTD with different metals exhibit different conformations and binding sites

**Figure 1:**
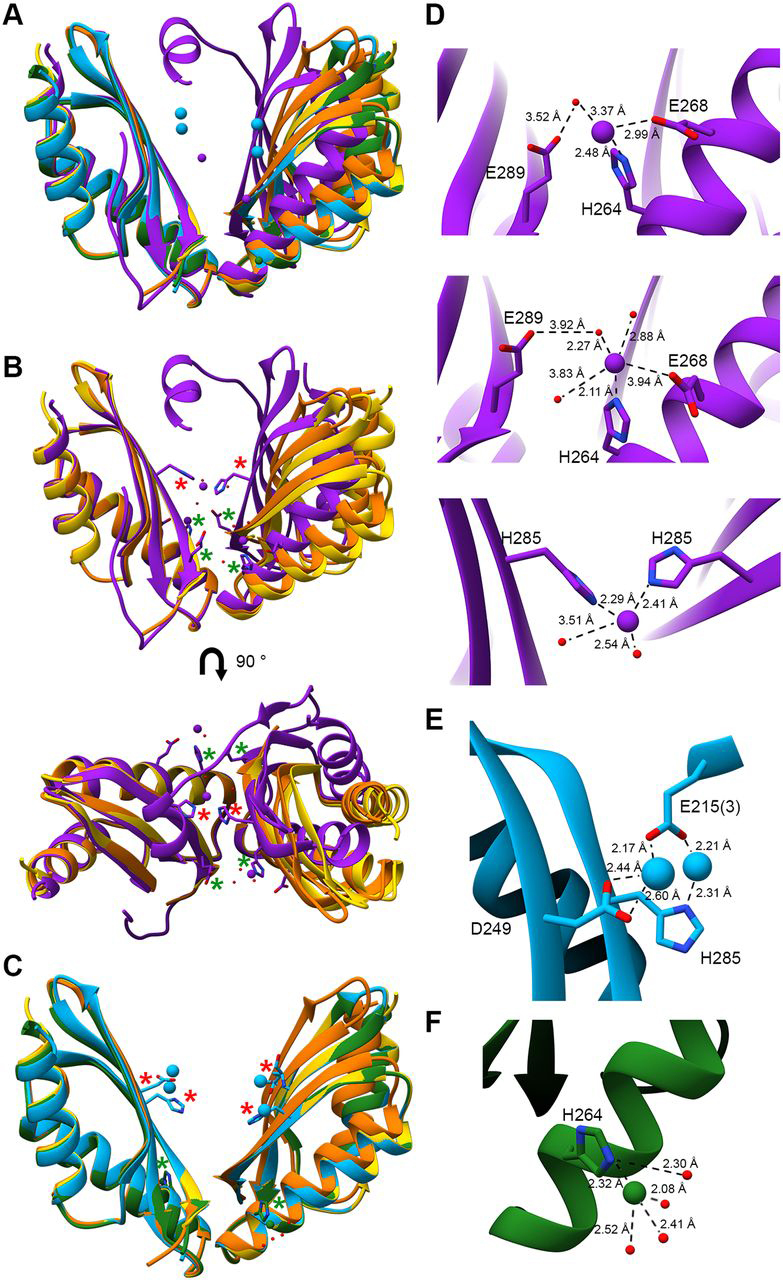
**Crystal structures of MamM CTD with different metal cations.***A*. the three MamM CTD bound structures (Cd^2+^ (PDB ID:6GMT), *blue*; Ni^2+^ (PDB ID: 6GMV), *green*; Cu^2+^ (PDB ID: 6GP6), *purple*) and the two apo structures (PDB ID: 3W5X in *yellow*, PDB ID: 3W5Y in *orange*), overlapped onto each other. Metal cations are presented for each bound structure in the same colors. *B*, apo forms and Cu^2+^-bound structures overlapped onto each other from two angles, showing the conformational change exhibited by the Cu^2+^-bound structure. The metal-binding residues are presented as *sticks* and pointed out with *asterisks* (CS residues in *red* and PS residues in *green*). *C*, apo forms, Ni^2+^-bound and Cd^2+^-bound structures overlapped onto each other, indicating no conformational changes between all structures. The metal-binding residues are presented as *sticks* and pointed up with *asterisks* (CS residues in *red* and PS residues in *green*). *D*, close-up of the Cu^2+^-bound structure binding sites (PSs, *upper* and *middle panels*; CS, *lower panel*). *E*, close-up of the Cd^2+^-bound structure binding site (of one monomer). (3) refers to a nonbiological-assembly monomer. *F*, close-up of the Ni^2+^-bound structure binding site (of one monomer). In (*D*), (*E*), and (*F*), the metals, metal-binding residues, metal-bound water molecules, and relevant bond distances are presented.

The crystal structure of apo MamM CTD has been previously solved in two space groups, both revealing the same monomeric fold and dimerization interface (PDB IDs: 3W5X and 3W5Y) (Fig. 1, *A*–*C*, see RMSDs in Table 1 and distances and dihedral angels in Table S4) (5). To better achieve an understanding of the conformational changes occurring during metal binding in MamM (5, 21), co-crystallization trials of MamM CTD (residues 215–318) with various divalent transition metal cations (Fe^2+^, Mn^2+^, Zn^2+^, Ni^2+^, Co^2+^, Cu^2+^, and Cd^2+^) under a range of different conditions were performed. Crystal structure models of MamM CTD were only obtained in the presence of three different metal cations: Cd^2+^, Ni^2+^, and Cu^2+^ (although crystals containing Zn^2+^ diffracted well, Zn^2+^ could not be detected in the electron density map). The structures with bound Cd^2+^ and Ni^2+^ indicate no conformational change both in their monomeric fold and their dimeric interface, as compared with the apo form (Fig. 1, *A* and *C*, Table 1, and Table S4). In contrast, the Cu^2+^-bound form reveals a clear change in the dimeric conformation to a tighter dimer interface, as has already been demonstrated in other CDF proteins and proposed for zinc-bound MamM CTD in solution using PELDOR spectroscopy (Fig. 1, *A* and *B*, Table 1, and Table S4) (21, 23). Furthermore, in this specific Cu^2+^-bound structure, most of the C-terminal tail of one of the monomers, which was not previously identified in the apo forms' electron density maps, is now resolved. The C-terminal residues (292–314) form a short β-strand that adds to the previously solved three-stranded β-sheet, resulting in a 4-stranded β-sheet, followed by an α-helix. The C-terminal helix is found in the space between the two monomers and forms a stabilizing bonding network with the adjacent monomer's residues. This nonsymmetric interaction may be due to crystal packing or some as yet unknown biological function. The α-helical tail stabilizes the CTD closed state and may interact with the TMD loops to also stabilize the transporter's transmembrane conformation in its activated state as could be seen in the recently determined structure of ZnT-8 (24). However, because CDFs homodimers have not previously revealed nonsymmetric characteristics, we propose that these C-terminal interactions may be locking the dimer in its closed state, resulting in the stabilized crystal packing.

**Table 1:** RMSDs between different CDFs' CTD dimeric structures

Protein form	MamM apo (3W5X)	MamM apo (3W5Y)	MamM Cu^2+^-bound (6GP6)	MamM Cd^2+^-bound (6GMT)
MamM apo (3W5X)	-	0.82	1.56	0.35
MamM apo (3W5Y)	-	-	1.60	0.89
MamM Cu^2+^-bound (6GP6)	-	-	-	1.62
MamM Cd^2+^-bound (6GMT)	-	-	-	-
MamM Ni^2+^-bound (6GMV)	0.18	0.91	1.58	0.44
CzrB apo (3BYP)	1.49	1.59	-	-
CzrB bound (3BYR)	-	-	1.43	1.64
EcYiiP bound (3H90)	-	-	1.33	1.19
SoYiiP bound (5VRF)^a^	-	-	-	-
MamB apo (5HO5)	1.21	1.20	1.32	1.26^b^
MamB bound (5HO1)	1.76	1.62	1.37	1.13


All RMSDs (given in Ångstroms) between the dimeric structures were calculated using the iterative magic fit tool of Swiss-PdbViewer 4.1.0.

a Satisfying fit directly between the dimeric MamM and SoYiiP structures could not be obtained.

b RMSD was calculated using the domain fragment alternate fit tool of Swiss-PdbViewer 4.1.0.

Closer inspection of the different structures' metal-binding sites confirms the identity of the previously proposed residues involved in metal binding (5, 21). The Cu^2+^-bound structure contains three binding sites: a central binding site (CS) in which one copper ion is bound by His-285 from both monomers and an additional two water molecules, and two “symmetric” peripheral binding sites (PSs), each of which binds one copper ion (Fig. 1, *A*, *B*, and *D*, see metal-ligand distances in Table 2). In both PSs, His-264 chelates the copper ion. In one of the PSs, three water molecules appear to stabilize the ion at this location, and one of them bridges between Glu-289 in the adjacent monomer and the metal ion. Glu-268 is also close to the copper ion at this site; however it does not appear to ligate directly or through a water molecule, whereas in the second PS, Glu-268 is much closer to the ion and participates directly in its chelation (Table 2). In this second PS (much like in the first), a water molecule also bridges between Glu-289 in the adjacent monomer and the metal ion. Because Glu-289 has previously been shown to affect MamM function (5), and because binding to the PSs appears to facilitate tighter dimeric packing (21), we propose that this water molecule plays an important role in the stabilization of the closed state.

**Table 2:** Coordination distances to ions in MamM CTD metal-bound crystal structures

Protein form	Glu-215 3^rd-^mer	Asp-249	His-285	His-264	Glu-289 to water	Glu-268	Water
Cu^2+^-bound (6GP6)	-	-	2.41^3^ 2.29^3^	2.48^1^ 2.11^2^	3.52*^a^*,^1^ 3.92*^b^*,^2^	2.99^1^ 3.94^2^	3.37*^a^*,^1^ 2.27*^b^*,^2^ 2.88^2^ 3.83^2^ 3.51^3^ 2.54^3^
Cd^2+^-bound (6GMT)	2.17^1^ 2.21^2^	2.44^1^ 2.60^1^	2.31^2^	-	-	-	-
Ni^2+^-bound (6GMV)	-	-	-	2.32	-	-	2.30 2.08 2.41 2.52


All distances were measured using UCSF Chimera (34).

For the Cu^2+^-bound structure, Glu-289 and the water molecule that coordinates it to the Cu^2+^ ion are marked as *^a^* for one peripheral site (Fig. 1*D*, *upper panel*) or *^b^* for the second peripheral site (Fig. 1*D*, *middle panel*).

For the Cu^2+^-bound structure, ^1^ relates to coordination of the Cu^2+^ ion in one peripheral site (Fig. 1*D*, *upper panel*), ^2^ relates to coordination of the Cu^2+^ ion in the second peripheral site (Fig. 1*D*, *middle panel*), and ^3^ relates to coordination of the Cu^2+^ ion in the central site (Fig. 1*D*, *lower panel*).

For the Cd^2+^-bound structure, ^1^ relates to coordination of one Cd^2+^ ion in the central site (Fig. 1*E*, *left ion*) and ^2^ relates to coordination of the second Cd^2+^ ion in the central site (Fig. 1*E*, *right ion*).

In the Cd^2+^-bound structure, which contains two bound metals per monomer (*i.e.* four metal sites in total), Asp-249 and His-285 each bind one cadmium ion in each monomer. Both cadmium ions are also chelated by Glu-215 from a nonbiological-assembly monomer (Fig. 1, *A*, *C*, and *E* and Table 2) and possess half-occupancy in the electron density map, suggesting that they may be bound alternately. Mutations of both Asp-249 and His-285 to alanine, either separately or together, were previously shown to affect the function of MamM CTD both *in vitro* and *in vivo* (5, 21). This Cd^2+^-bound structural model indicates that both residues are able to bind metals, although this binding alone does not appear sufficient to induce the same conformational change as observed with Cu^2+^. Our previous observation of conformational change toward a closed stable state upon Zn^2+^ binding was only induced upon metal binding to both the PSs. Hence, binding of Cd^2+^ to the CS alone (in agreement with Zn^2+^ binding to the CS alone (21)) is not expected to cause a distinct conformational change.

In the Ni^2+^-bound structure, which only contains one bound metal per monomer in the peripheral site, this nickel ion is bound to His-264 and to water molecules, possibly implying a nonspecific interaction of the Ni^2+^ ion with the histidine (Fig. 1, *A*, *C*, and *F* and Table 2). However, specific chelation to this histidine rather than to other metal-binding residues might suggest a higher affinity for nickel, whereas its location in the protein periphery could also suggest it plays an important role in attracting the metal ions. This proposal is supported by previous studies that demonstrated the impact of His-264 on MamM function (5) and the new Cu^2+^-bound structure.

Interestingly, all the metal-dependent crystallographic binding sites can be well-explained by the metals' propensities to be bound by specific amino acids: Cu^2+^ has the highest affinity for histidine, followed by Ni^2+^ with Cd^2+^ having the lowest affinity, whereas the aspartate and glutamate binding tendencies show the opposite trend (7). Overall, the MamM CTD metal-bound crystal structures confirm the previously proposed binding sites and reveal diversity among the different metals' chelation. Because each metal is bound by different residues and only the Cu^2+^-bound structure reveals the apparent conformational change, these crystallographic results would indicate that each metal exhibits a different binding mode. These binding modes may well underpin the prevention of the correct transport activation via such altered conformational changes and thus may distinctly regulate the overall protein.

### Dimer packing of the MamM CTD Cu^2+^-bound structure is less tight compared with other CDF CTD bound structures

**Figure 2:**
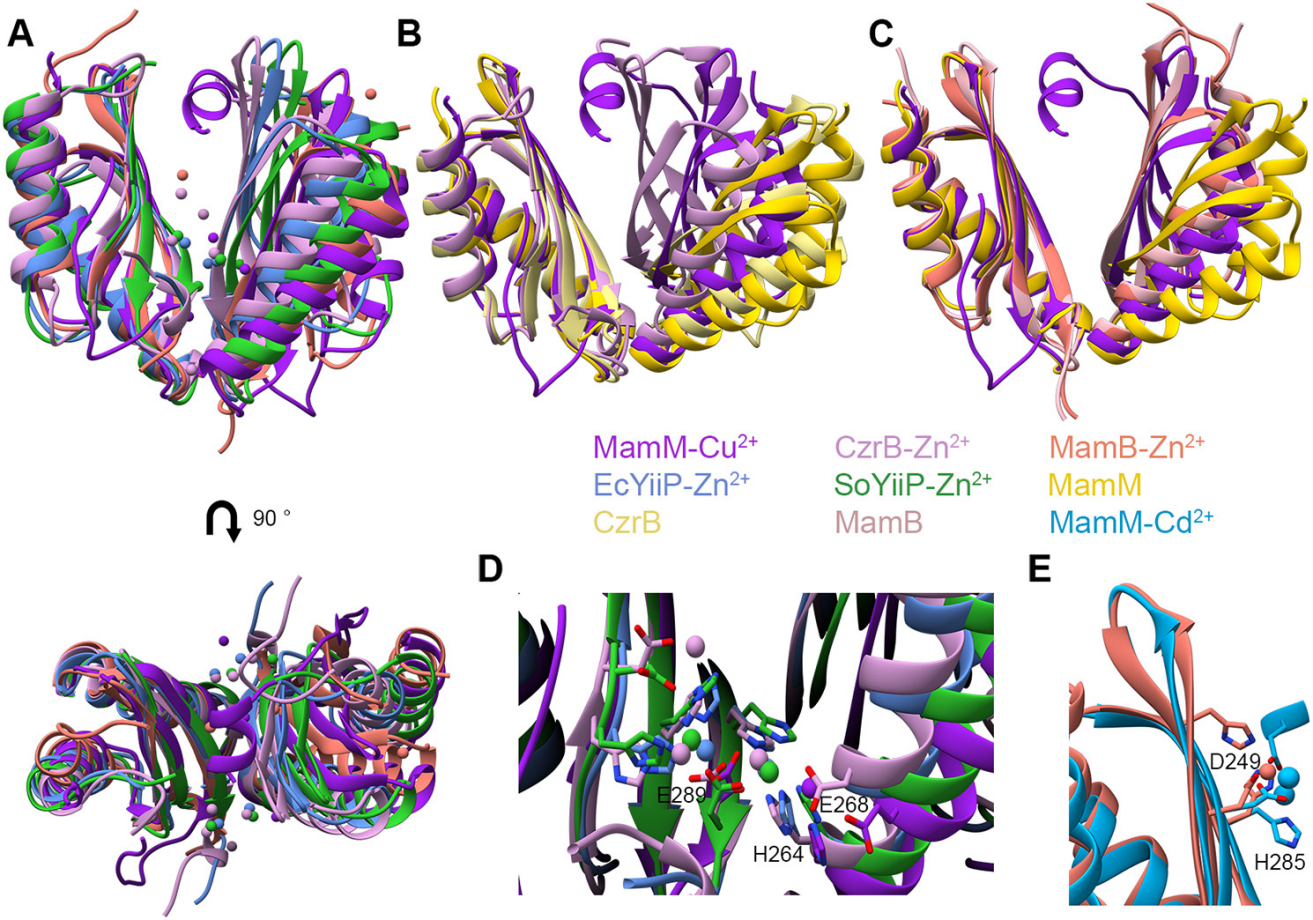
**Comparison of the conformational changes and binding sites of different bacterial CDFs' CTDs.** Colors legend: MamM Cu^2+^-bound (PDB ID: 6GP6, *dark purple*), CzrB Zn^2+^-bound (PDB ID: 3BYR, *light purple*), MamB Zn^2+^-bound (PDB ID: 5HO1, *salmon*), EcYiiP Zn^2+^-bound (PDB ID: 3H90, *cornflower blue*), SoYiiP Zn^2+^-bound (PDB ID: 5VRF, *green*), MamM apo (PDB ID: 3W5X, *dark yellow*), CzrB apo (PDB ID: 3BYP, *light yellow*), MamB apo (PDB ID: 5HO5, *light pink*), MamM Cd^2+^-bound (PDB ID: 6GMT, *sky blue*). *A*, comparison between all bound CDF CTDs structures at two angles: MamM Cu^2+^-bound, MamB, CzrB, EcYiiP, and SoYiiP (all Zn^2+^-bound). Metal cations are presented for each bound structure in the same colors. *B*, CzrB exhibits much larger conformational change between apo and bound states compared with MamM; MamM apo, MamM Cu^2+^-bound, CzrB apo, and CzrB Zn^2+^-bound structures are overlapped. *C*, MamB exhibits no conformational changes between the bound and apo states and shows tighter packing compared with MamM apo form; MamM apo, MamM Cu^2+^-bound, MamB apo, and MamB Zn^2+^-bound structures are overlapped. *D*, peripheral site binding residues are conserved between MamM, CzrB, and YiiP proteins. Metal cations are presented for each bound structure in the same colors; metal-binding residues are presented as *sticks*. The presented residue labels refer to MamM. *E*, central site binding residues are conserved between MamM and MamB proteins. Metal cations are presented for each bound structure in the same colors; metal-binding residues are presented as *sticks*. The presented residue labels refer to MamM.

Previously, a number of CDF protein CTD structures were solved in their apo state, their Zn^2+^-bound state (YiiP from *Escherichia coli* and YiiP homolog from *Shewanella oneidensis*, EcYiiP and SoYiiP respectively, and human ZnT-8), or in both apo and Zn^2+^-bound states (MamB and CzrB) (1, 2, 3, 4, 23, 24, 25) (Fig. 2*A*). According to these solved structures, the only protein to exhibit a distinct closure of the V-shape upon metal binding compared with its apo state is CzrB (PDB IDs: 3BYP and 3BYR) (23). The bound state of CzrB exhibits a larger degree-of-closure than that of the MamM CTD Cu^2+^-bound state, demonstrated by the larger differences in the distances and the dihedral angles between equivalent residues in the apo and closed states (Fig. 2*B*, Table 1, and Table S4). This is due not only to a tighter closed state but also to a slightly more open apo form compared with those of MamM CTD. MamB, another CDF protein from MTB, exhibits very little conformational change between the apo and Zn^2+^-bound states (25). However, the degree-of-closure in both MamB CTD structures is larger than that of MamM CTD apo structures and is similar to that of the MamM Cu^2+^-bound structure (PDB IDs: 5HO1 and 5HO5; Fig. 2*C*, Table 1, and Table S4). The structures of the entire CDF proteins EcYiiP, SoYiiP, and human ZnT-8 (including both the CTD and TMD) have also been solved with the CTD in the Zn^2+^-bound state (PDB IDs: 3H90, 5VRF, and 6XPD) (2, 4, 24). The CTD structures are highly conserved in the high-resolution YiiP structures, and the degree-of-closure is also greater than that of the MamM Cu^2+^-bound state (Fig. 2*A*, Table 1, and Table S4). However, this cannot be directly compared with the overall conformational change exhibited by MamM between apo and bound states, because the apo structures of EcYiip and SoYiiP are not currently available and because ZnT-8 is stabilized in a permanent closed conformation via its C and N termini. In conclusion, the MamM CTD Cu^2+^-bound structure shows moderate dimerization closure compared with other CDF bound proteins, as was also proposed in our previous study of Zn^2+^-binding and associated conformational change in solution (21). This may be due to the presence of an additional CTD terminal helix within MamM, which is not found in other CDF protein structures and which potentially extends the interdimer gap. However, there is variation in the degree-of-closure between the CDF CTD's of other proteins, suggesting that some degree of flexibility in the CTD conformational changes between the different CDF family members exists. Noticeably, this also depends on the CTD-TMD interface that occurs mainly between the CTD and TMD loops. This interface can vary between the different proteins and thus affect the degree-of-closure exhibited by each individual protein's CTD.

The binding sites present in all MamM CTD metal-bound structures are consistent with those of other CDF protein binding sites. MamM PSs are composed of His-264 (Cu^2+^-bound and Ni^2+^-bound structures) and Glu-289 via a bridging water molecule (Cu^2+^-bound structure). In CzrB, six zinc ions are bound to the CTD through three sets of symmetric binding sites (Fig. 2*D*) (23). Two Zn^2+^ ions are bound in symmetric PSs each by His-258 (from one monomer), Glu-282, and His-280 (from the second monomer), where residues His-258 and Glu-282 are homologous to His-264 and Glu-289 in MamM PSs (His-280 is homologous to Ser-287 in MamM). Two additional Zn^2+^ ions are symmetrically bound by Glu-282, and additional residues of the same monomer that are not homologous to any of the MamM CTD binding residues but to residues that do not usually participate in metal binding. These residues also participate in the binding of the last two symmetric Zn^2+^ ions. Overall, this protein-Zn^2+^ interaction network stabilizes Zn^2+^ binding in the peripheral sites, which are the only sites to exhibit binding by residues in both monomers, indicating that the PSs are important for conformational change in CrzB. In the YiiP bound structures, two symmetrical pairs of Zn^2+^ ions are bound in the same locations and by homologous residues to the first two Zn^2+^ pairs in CzrB (Fig. 2*D*) (2, 4), further highlighting the importance of the peripheral sites. Although the Glu-289 residue in MamM does not chelate the Cu^2+^ directly in the crystal structure, Zn^2+^ is bound by the homologous residues to Glu-289 in the crystal structures of the other Zn^2+^-bound CDF proteins. Furthermore, Glu-289 is crucial for proper function *in vitro* and *in vivo* (5). Thus, it is clear that Glu-289 in MamM plays an important role in metal binding for biological function.

The MamM Cu^2+^-bound structure also involves one Cu^2+^ ion bound centrally by His-285 from each monomer and an additional two water molecules. Homologous to His-285 of MamM is residue His-283 in MamB, involved in the chelation of Zn^2+^. In MamB, one Zn^2+^ ion is coordinated in each monomer by residues His-283, Asp-247 (homologous to Asp-249 in MamM), and His-245 (found in the same location as Trp-247 in MamM) (Fig. 2*E*) (25). Whereas the MamM Cu^2+^-bound structure involves only His-285 (but from both monomers, together binding a single copper ion), the Cd^2+^-bound structure exhibits binding more similar to that of MamB. In the Cd^2+^-bound structure, no cadmium ions are bound to the PSs, and residues of MamB that are homologous to the MamM PSs would not afford effective metal cation binding. Additionally, both metal-bound structures exhibit no conformational changes compared with their apo forms. However, unlike the MamB Zn^2+^-bound structure, Cd^2+^-bound MamM includes two Cd^2+^ ions per monomer (one bound by His-285 and the other by Asp-249). Nonetheless, because these Cd^2+^ ions possess only half-occupancy in the electron density map, on average just one ion is bound in each monomer, as is the case with MamB. When considering all CDF metal-bound structures, a central site exists only in MamM and MamB, and mutations of these CS residues in both proteins were shown to lead to lower functionality both *in vivo* and *in vitro* (5, 25). Noticeably, copper binding in a unique coordination in the CS facilitates subsequent binding to the PS (as was shown for Zn^2+^ (21)), or vice versa. Overall, the analysis of all known structures suggests that the CS and PSs bind different metals distinctively in MamM, strengthening the previous observation that only when metals are bound to the PSs can the conformational changes effectively occur.

### MamM CTD shows metal-type-dependent change in tryptophan fluorescence signal

**Figure 3:**
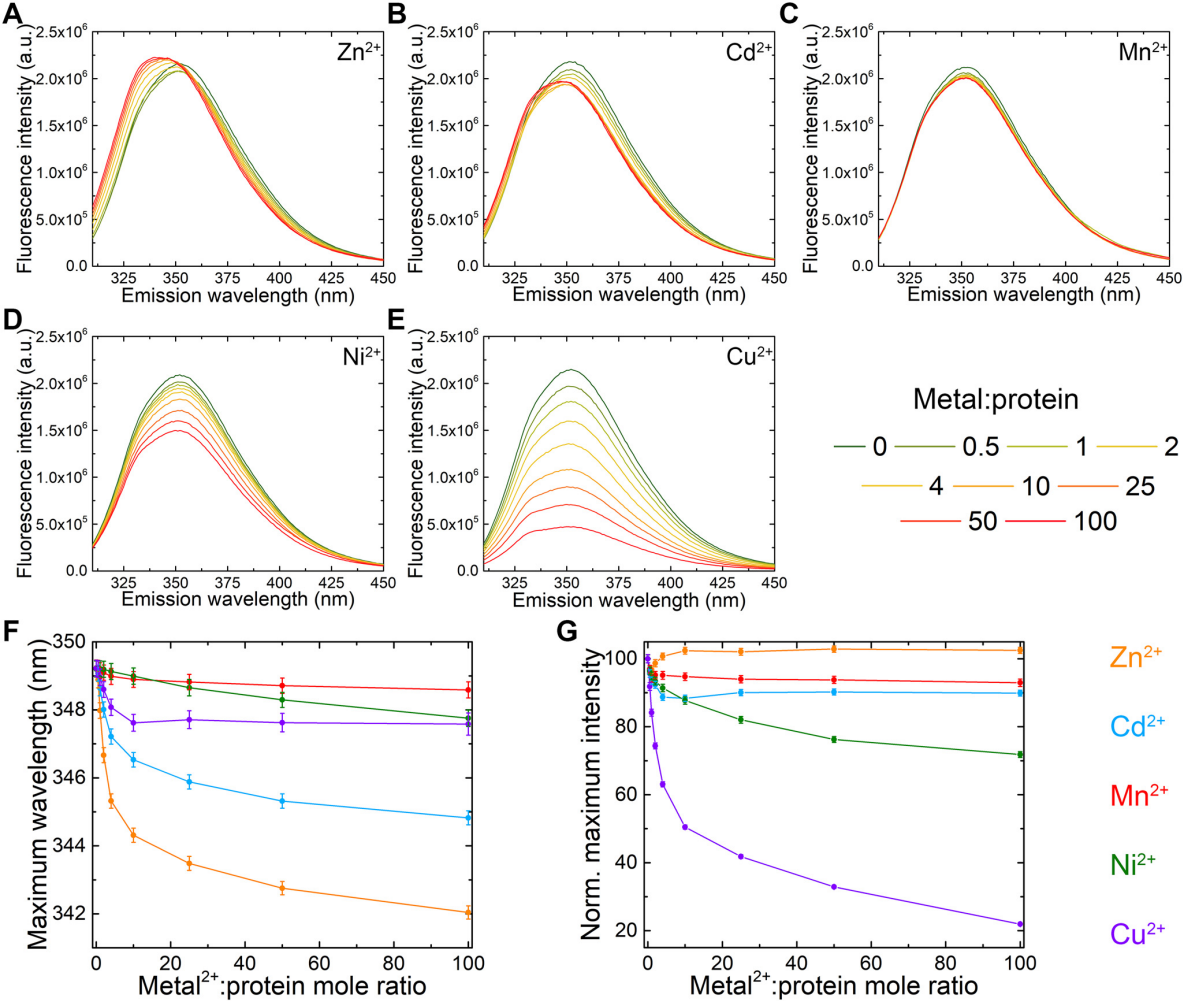
**Fluorescence spectral scans of MamM CTD with different metals.** All emission spectra of 5 µm MamM CTD were titrated using different metal solutions to reach different MamM CTD:Metal^2+^ ratios (intensities were normalized because of the change in MamM CTD concentration). Samples were measured at λ_ex_ 297 nm, and the emission spectrum for each metal concentration was recorded at 310–450 nm and presented as arbitrary units (*a. u.*). Each presented spectrum is the average of three independent measurements. *A*, emission spectra of MamM CTD with different Zn^2+^ concentrations. *B*, emission spectra of MamM CTD with different Cd^2+^ concentrations. *C* emission spectra of MamM CTD with different Mn^2+^ concentrations. *D*, emission spectra of MamM CTD with different Ni^2+^ concentrations. *E*, emission spectra of MamM CTD with different Cu^2+^ concentrations. *F*, λ_max_ as function of Metal^2+^:MamM CTD mole ratio. *G* normalized fluorescence intensity compared with no Metal^2+^ as function of Metal^2+^:MamM CTD mole ratio. In (*F*) and (*G*), Zn^2+^ is presented in *orange*, Cd^2+^ in *blue*, Mn^2+^ in *red*, Ni^2+^ in *green*, and Cu^2+^ in *purple*, and *error bars* are shown within the symbols.

Crystal structures often only provide limited and sometimes biased information on the conformation of proteins. Hence, to further characterize MamM CTD binding to different metal cations, we have investigated these interactions in solution. Each MamM CTD monomer contains one tryptophan residue, Trp-247, juxtaposed to the CS. When metal cations bind to MamM CTD, it is proposed that the monomers approach each other, and hence the tryptophan environment will be changed, which should lead to a shift in the emission spectrum of the tryptophan residue when excited at the tryptophan-specific wavelength (λ = 297 nm). In the case of paramagnetic metals, their binding near this tryptophan will cause the tryptophan fluorescence intensity to be quenched. We have shown previously that the addition of Zn^2+^ causes a blue shift in the emission spectrum of MamM CTD and that the addition of the paramagnetic Fe^2+^ causes signal quenching (5, 21), indicating that MamM CTD binds both cations. In this study, we have titrated further metal cations (Cd^2+^, Ni^2+^, and Cu^2+^, in which their bound crystal structures were detected, and Mn^2+^, which was shown to be transported by numerous CDF proteins (7, 8)) into MamM CTD solution samples and measured the concentration-dependent changes in the tryptophan emission spectra (ratios of 0:1 to 100:1 metal:protein, Fig. 3). As shown previously for Zn^2+^ (Fig. 3*A*, adopted from (21)), in the case of the diamagnetic Cd^2+^, a shift in maximum wavelength could be observed (Fig. 3*B*), suggesting that the binding of Cd^2+^ leads to a change in the tryptophan environment. However, although the spectral shift is accompanied by an increase in intensity for Zn^2+^, the intensity decreases when Cd^2+^ is added and the blue shift is smaller (Fig. 3, *F* and *G*). The Cd^2+^-bound crystal structure suggests no conformational change compared with the apo form, whereas it was previously shown that the addition of Zn^2+^ causes a tight closure of the CTD (21). Because cadmium is much bigger than zinc, Cd^2+^ binding might restrict the ability of the dimer to pack tighter, which could result in a smaller shift and the difference in the fluorescence intensity observed for Cd^2+^. Alternatively, it is also possible that the Cd^2+^ ions, which are bound in close proximity to Trp-247, simply influence the tryptophan fluorescence signal differently. Mn^2+^, Ni^2+^, and Cu^2+^ are all paramagnetic; hence their binding to the CTD would be expected to cause observable fluorescence quenching leading to a stronger signal than their binding-dependent spectral shift. However, the addition of Mn^2+^ only reveals minor changes in the fluorescence intensity (Fig. 3, *C* and *G*) and in the maximum wavelength (Fig. 3*F*), suggesting that Mn^2+^ cannot bind to the CTD in a way that would cause a change in the fluorescence spectra. In contrast, Ni^2+^ and Cu^2+^ demonstrate clear metal concentration–dependent quenching (Fig. 3, *D*, *E*, and *G*) and a slight spectral shift (Fig. 3*F*), with Cu^2+^ exhibiting stronger quenching and a larger shift. Overall, these results indicate that MamM CTD can bind all the examined metals with the exception of Mn^2+^.

### MamM CTD exhibits metal-type–dependent binding thermodynamics

**Figure 4:**
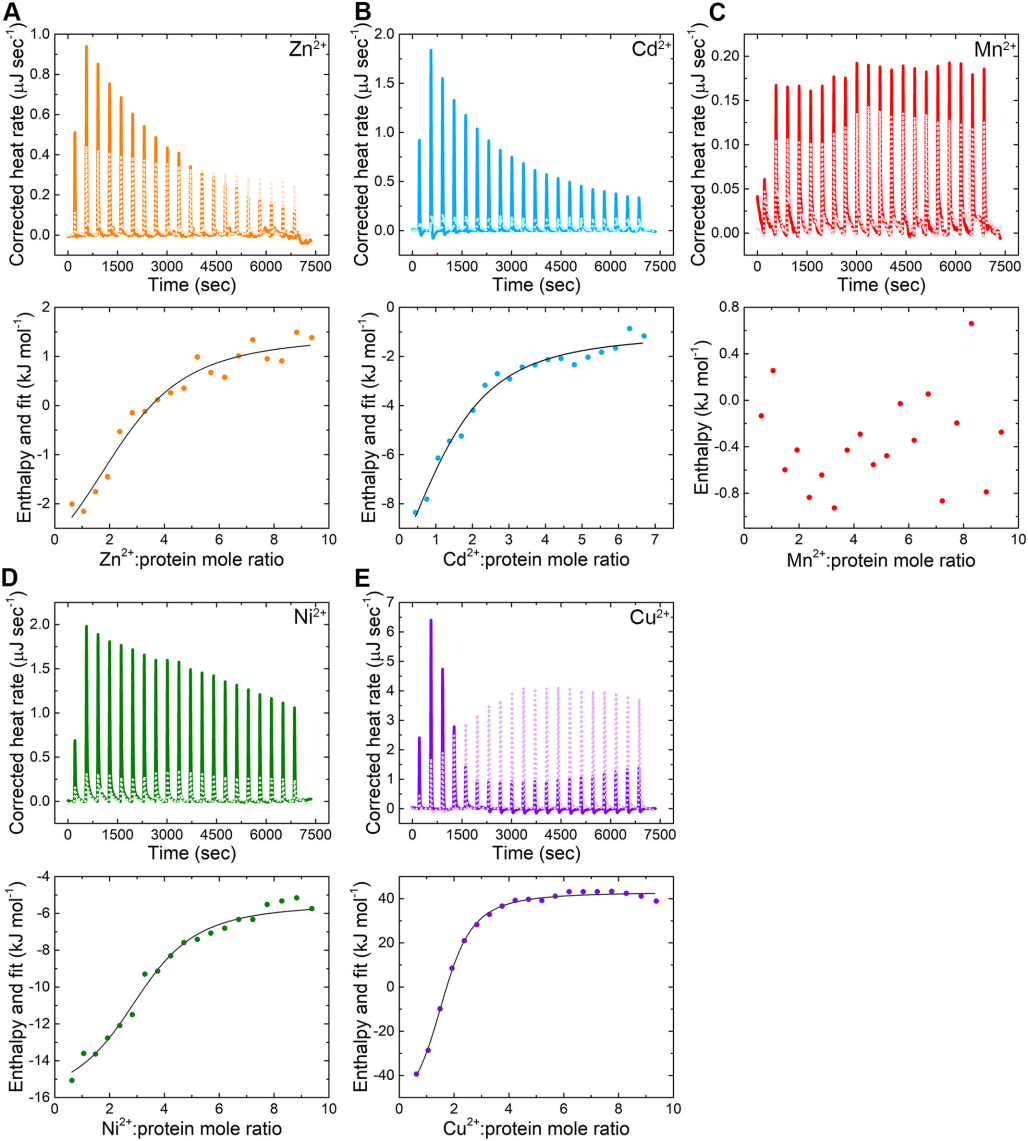
ITC titration measurements of MamM CTD-metals interactions, with (*A*) Zn^2+^, (*B*) Cd^2+^, (*C*) Mn^2+^, (*D*) Ni^2+^, and (*E*) Cu^2+^. *Top panels*: Corrected heat rate curves for a representative titration of MamM CTD with each metal as a function of time. For each metal, the heat flow curve of the control is given as a reference (metal titration into buffer, *dotted line in brighter color*). *Bottom panels*: Titration data after peak integration as a function of Metal^2+^:MamM CTD mole ratio, and data fitting for all metals except Mn^2+^. See Table 3 for binding parameters.

To accurately calculate the affinity of each metal to MamM CTD, other thermodynamic parameters, and the number of metal cations that can bind to the CTD, ITC measurements were performed (Fig. 4). Here Cd^2+^, Ni^2+^, Cu^2+^, and Mn^2+^ were titrated into MamM CTD solution samples. All ITC parameters are summarized in Table 3, including those for previously titrated Zn^2+^ (21) (note: metal binding to MamM CTD requires a slightly basic pH because of the metal chelation by histidine residues; therefore titration of Fe^2+^ leads to iron precipitation and hence its binding parameters cannot be accurately determined). ITC results indicate that all metals except Mn^2+^ induce a heat change when titrated into a MamM CTD solution. Hence taken together with the fact that Mn^2+^ revealed no fluorescence shift, we propose that MamM CTD is unable to bind Mn^2+^.

**Table 3:** Thermodynamic parameters of MamM CTD binding to different metals

Parameter	Zn^2+^^a^	Cd^2+^	Ni^2+^	Cu^2+^
*K_d_* (µm)	31.50 ± 3.40	48.39 ± 2.61	17.71 ± 3.35	6.00 ± 3.83
N	2.80 ± 0.24	1.23 ± 0.08	3.26 ± 0.19	1.56 ± 0.15
ΔH (kJ mol^−1^)	−4.20 ± 0.10	−13.08 ± 1.56	−7.78 ± 2.38	−71.80 ± 23.59
ΔG (kJ mol^−1^)	−25.71 ± 0.26	−24.64 ± 0.13	−27.17 ± 0.48	−30.13 ± 1.59
ΔS (J mol^−1^ K^−1^)	72.15 ± 0.98	38.76 ± 4.81	65.03 ± 9.59	−136.72 ± 83.76


ITC data were fitted to independent + blank (constant) models in the TA NanoAnalyze Data Analysis software.

Each parameter represents three independent measurements after control reduction (metal at the same concentration titrated into buffer solution).

The buffer used for all titrations: 10 mm Tris-HCl, pH 8.0, and 150 mm NaCl.

a The data are taken from (21).

The *K_d_* values determined vary from 6–48 µm, with Cu^2+^ having the greatest affinity followed by Ni^2+^, Zn^2+^, and Cd^2+^ (Table 3). As revealed in all metal-bound crystal structures and previous *in vivo* studies (5), histidine residues are the major components for metal binding. Again, the larger size of the Cd^2+^ ion may explain its low affinity because its coordination may be less preferred; however, a lower propensity to bind histidine residues compared with the other metals (7) may also result in a lower affinity to proteins with multiple histidine-containing binding sites such as MamM CTD. Cu^2+^ has the highest propensity to bind histidine, which clearly explains its high affinity to MamM. Ni^2+^ also exhibits a high propensity for histidine binding compared with Zn^2+^ and Mn^2+^ (7). Although all binding reactions are exothermic (Table 3), Cu^2+^-binding displays much lower enthalpy, which means it is favored in terms of the heat release. Moreover, whereas Ni^2+^ and Zn^2+^, but also Cd^2+^, display high entropy, the entropy of Cu^2+^ binding is negative, suggesting that its binding results in a more ordered state of the cations compared with its free state in solution. Summarizing all of the above, the negative Gibbs free energy of the binding reactions implies that all metal-binding reactions are preferred to the apo state (excluding Mn^2+^), yet MamM CTD shows clear preferences for the binding of specific metals over others.

The number of metal cations bound per dimer is also given by the ITC measurements and varies significantly between metals (Table 3). For Zn^2+^ and Ni^2+^, ∼3 cations are bound per dimer, in agreement with the Cu^2+^-bound structure. The Ni^2+^-bound crystal structure exhibits two cations per dimer in the PSs. However, it shows no conformational changes when compared with the apo state, and binding appears to be nonspecific; disagreements between the metal-bound structure and ITC results can therefore be expected.

For Cd^2+^ and Cu^2+^, lower numbers of metal cations bound per dimer are observed: 1.23 ± 0.08 for Cd^2+^ and 1.56 ± 0.15 for Cu^2+^. The numbers of metals observed in metal-bound structures are clearly not in agreement with these ITC observations, which are much lower. The Cd^2+^-bound structure reveals four metal-binding sites per dimer; however, the low occupancy observed in the electron density map would suggest that actually only two cations are bound at any one time. Moreover, binding in the crystal structure is mediated by a glutamate residue from a third monomer (from an adjacent unit cell), which may simply be a crystallographic artifact not necessarily expected to be observed in solution. Nevertheless, although the tryptophan-fluorescence scans suggest otherwise, this metal-bound structure does not exhibit any conformational change as also observed for the Ni^2+^-bound structure. Hence, the ∼1 Cd^2+^ ion bound per dimer determined from the ITC results suggests that binding at the CS is of a different nature from that displayed in the crystal structure.

The unexpectedly low number of Cu^2+^ ions bound in solution, as compared with the crystal structure, could have several explanations. It is quite possible that Cu^2+^ binding to the CS is silent in terms of the heat change (as was also shown previously for Zn^2+^-binding to the CS (21)); hence the thermodynamic parameters may relate only to the binding of two ions to the PSs (1.56 ∼ 2, and thus three Cu ions in total). Alternatively, in solution only one ion might bind at the CS, and none at the PSs (then 1.56 ∼ 1, in clear disagreement with the structure). However, the opposite may also be true, in which two ions are bound to the PSs and none to the CS (then again, 1.56 ∼ 2, and thus two overall, again in disagreement with the structural data). Because binding to the PSs has previously been shown to be the only cause of conformational change in MamM and other CDF CTDs, one would expect that if Cu^2+^ only binds to the CS, then no conformational changes should occur in solution, and that if Cu^2+^ only binds to the PSs, then a tighter conformation compared with the apo form would be detected in solution. The large tryptophan-fluorescence signal quenching demonstrated by titration of Cu^2+^ into MamM CTD indicates that the copper ions are bound in close proximity to Trp-247, hinting at an occupied CS. However, the tryptophan-fluorescence resolution is not sufficient to distinguish between these proposed options.

### EPR and PELDOR spectroscopy observe metal-bound MamM CTD conformational changes

**Figure 5:**
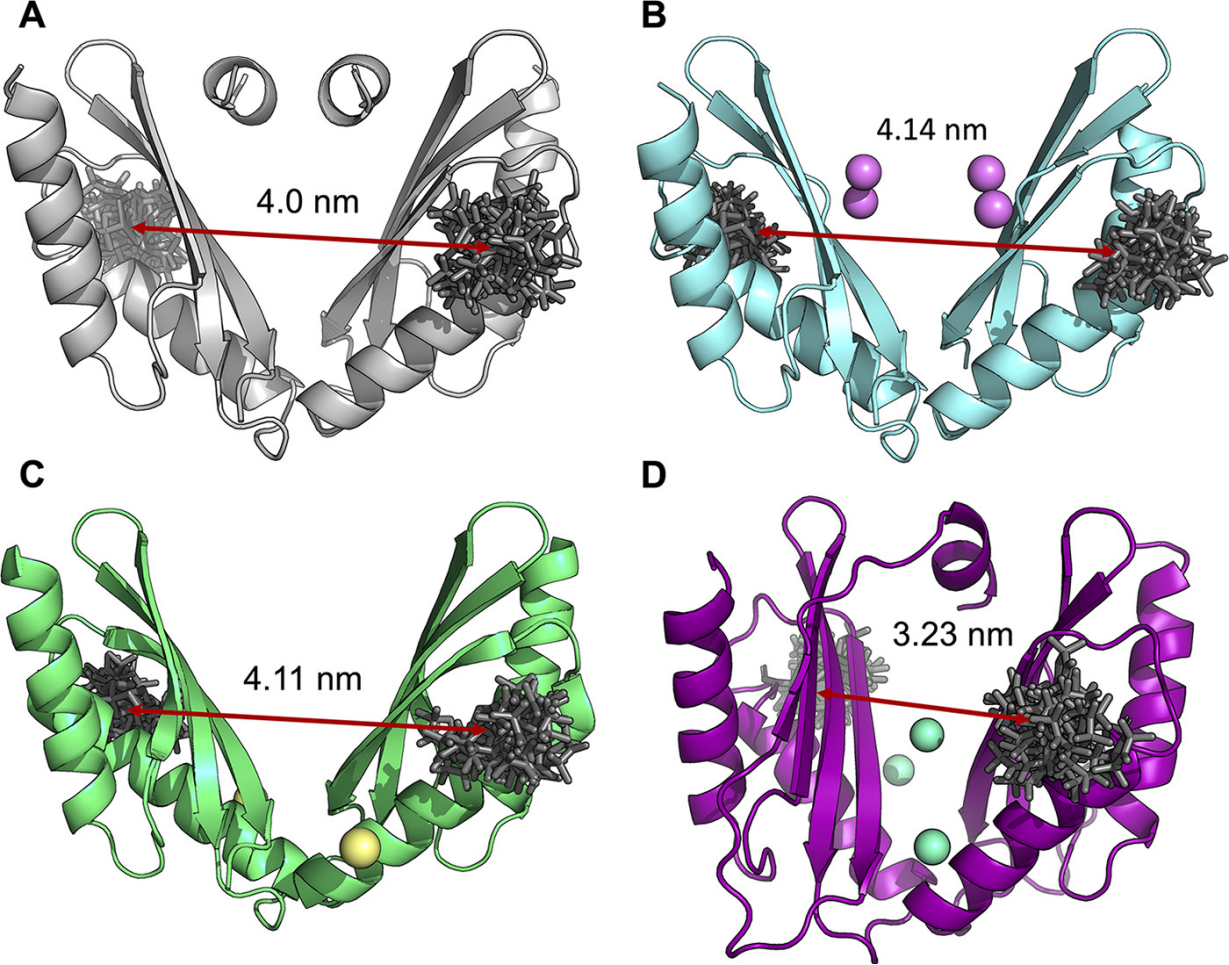
**MamM CTD structural models built using the published crystal structures.** Cartoon representation of (*A*) dimeric apo MamM CTD C267S (PDB ID: 6G55), (*B*) Cd^2+^-bound MamM CTD (PDB ID: 6GMT), (*C*) Ni^2+^-bound MamM CTD (PDB ID: 6GMV), and (*D*) Cu^2+^-bound MamM CTD (PDB ID: 6GP6) with the calculated rotamer libraries of MTSL attached to position Cys-275 shown as *sticks*, and with metal ions shown as *spheres*. *In silico* modeling of MTSL rotamer libraries and calculation of the average inter-spin distance was performed using MMM, a Matlab©-based open-source modeling toolbox.

**Figure 6:**
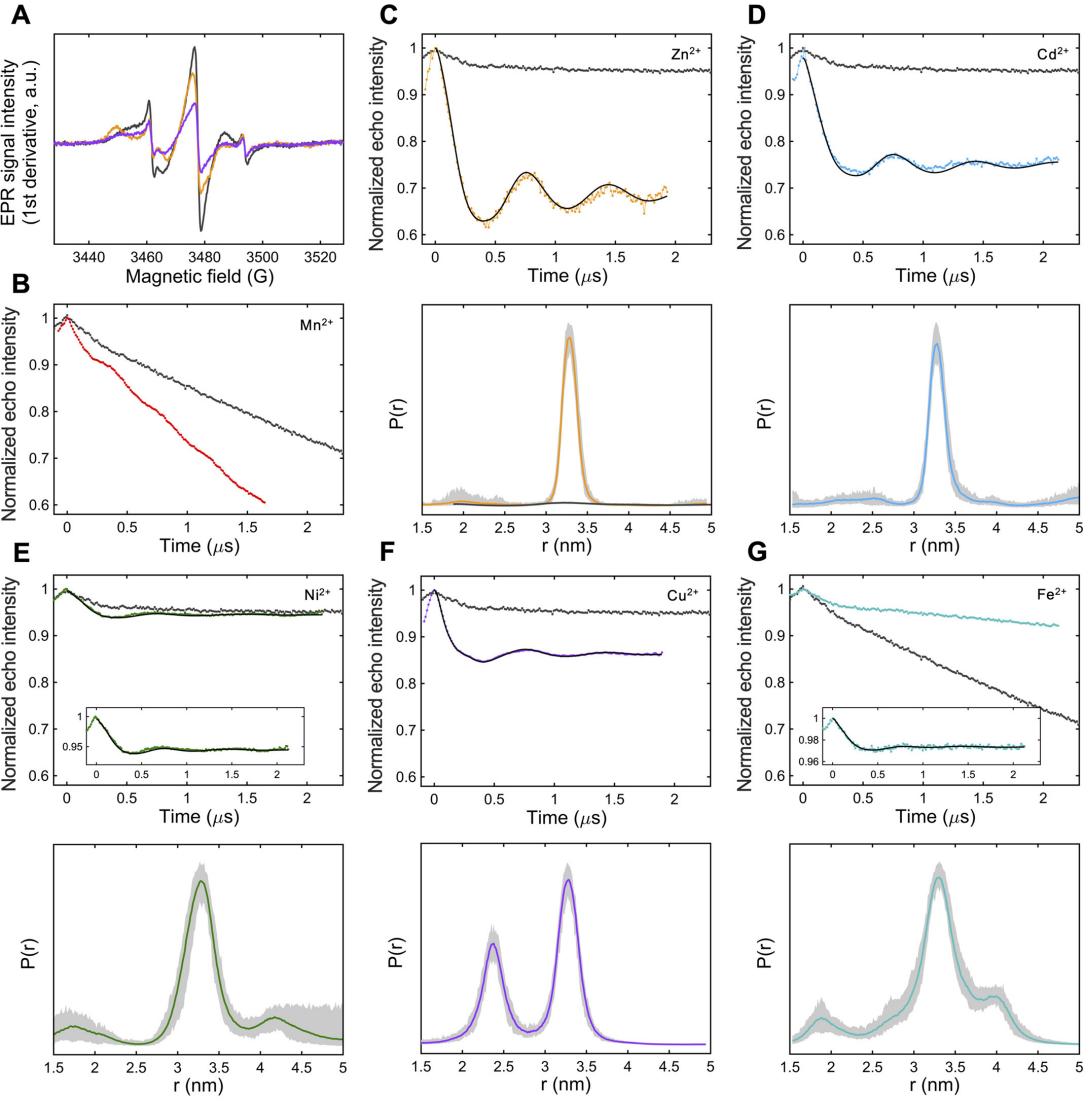
Room temperature continuous-wave EPR measurements (*A*) performed on apo MamM C267S_SL_ (*gray*), C267S_SL_-Zn^2+^ (*orange*), and C267S_SL_-Cu^2+^ (*purple*) revealing quenching of the EPR signal intensity upon addition of Cu^2+^ as compared with both the apo and Zn^2+^-bound states. *B*–*G*, four-pulse PELDOR experiments of doubly spin-labeled MamM CTD C267S, containing Cys-275 as the labeling position, for the measurement of inter-spin label distances. Results were obtained for samples incubated with a range of divalent transition metal ions, including Mn^2+^ (*B*), Zn^2+^ (*C*), Cd^2+^ (*D*), Ni^2+^ (*E*), Cu^2+^ (*F*), and Fe^2+^ (*G*). *Top panel*: The normalized background-corrected dipolar evolution of metal-bound samples (*bright colors*) presented together with the apo spin-labeled protein (*gray*). Exceptions: For both Mn^2+^ and Fe^2+^, the nonbaseline-corrected time traces are shown to clearly demonstrate the change in decay caused by specific *versus* nonspecific metal binding. *Bottom panel*: normalized distance distributions (where appropriate) of metal-bound C267S_SL_. The distance distribution of apo C267S_SL_ is also shown for reference, plotted against the Zn^2+^-bound results and shown in *dark gray*. Standard deviations in the form of 95% confidence intervals are also given in each case and represented by *light gray* areas around the lines. Fitting of all dipolar evolutions and subsequent distance distributions were calculated with DEERNet.

To probe divalent metal binding and the associated conformational changes reflected by intra-molecular distance, site-directed spin labeling was employed in combination with both continuous-wave EPR (cw-EPR) and pulsed electron-electron double resonance (PELDOR) spectroscopic studies (21). These methods provide a wealth of dynamic and structural information via observations of both changes in the local environment of an attached exogenous spin label elucidated by cw-EPR and the determination of long-range (typically between 2–8 nm) distance measurements and distance distributions afforded by PELDOR spectroscopy (26). We have previously reported (21) that using only one of the two intrinsic MamM CTD cysteine residues simplifies EPR measurements; hence the studies reported here use the same C267S variant of MamM CTD, in which only the Cys-275 site is available for labeling. *In silico* analysis using Multiscale Modelling of Macromolecules (MMM), a Matlab©-based open-source modeling toolbox (27), with both the apo WT and C267S CTD crystal structures (PDB IDs: 3W5Y and 6G55, respectively) predicts an average inter-spin distance of 4.0 nm between the labeled cysteines (Cys-275) in each protomer (Fig. 5*A*) (21).

cw-EPR studies at X-band (9 GHz) of complex dynamic proteins afford the observation of changes in the local environment of an attached exogeneous spin label and the overall mobility of the protein in different states. Studies were undertaken to ascertain what limiting effects, if any, the addition of zinc and copper metals (first-row transition metals to which the protein has a lower and higher affinity, respectively) has on the local spin label environment of C267S_SL_. Indeed, cw-EPR spectra of these metal-bound C267S_SL_ samples clearly differ substantially from those of the apo protein (Fig. 6*A*). The dynamics exhibited by spin labels attached to a protein have three main contributions: 1) the rotational correlation time of the protein itself; 2) the backbone motions of the protein at the point of spin label attachment; and 3) the localized intrinsic motion of the spin label. The latter two modes of motion are dominating factors in the line shapes of cw-EPR spectra at X-band, in particular their spectral broadness. Binding of metal ions to the protein resulting in conformational change affects these modes of motion, hence resulting in different spectral line shapes observed in Fig. 6*A*.

Our previous PELDOR studies of spin-labeled C267S MamM CTD (C267S_SL_), undertaken on apo and Zn^2+^-bound forms of the protein, afforded a direct comparison of the inter-spin distances giving insight into the conformational changes associated with Zn^2+^ binding (21). Although results for the apo protein clearly indicated that no single conformation is adopted, a very narrow distance distribution upon addition of Zn^2+^ (forming C267S_SL_-Zn^2+^) with a mean distance of 3.3 nm was interpreted as a change from a broad dynamically labile state of apo C267S (Fig. 6*C*) to a single closed conformation upon Zn^2+^-binding. Further, stoichiometric Zn^2+^ titrations, followed by PELDOR, suggested a maximum binding of 3–4 Zn^2+^ ions per dimer, in good agreement with ITC experiments (21). Here we have undertaken further cw-EPR and PELDOR studies on C267S_SL_ incubated with a range of divalent metal cations, including Cd^2+^, Ni^2+^, Cu^2+^, Mn^2+^, and Fe^2+^ (the natural substrate of MamM). Overall, our results indicate that although most PELDOR traces and associated distance distributions in the presence of divalent metal ions do show distinct differences from the apo protein, not all metal ion effects are equivalent.

The addition of both CdCl_2_ and NiCl_2_ to C267S_SL_ (×5 excess, forming C267S_SL_-Cd^2+^ and C267S_SL_-Ni^2+^, respectively) results in a distinct change in the PELDOR time trace obtained as compared with the apo case. A single and quite narrow distance distribution is predominant in both cases with an average distance of 3.3 nm (Fig. 6, *D* and *E*) and is very similar to the distribution obtained previously with Zn^2+^ (Fig. 6*C*, adopted from (21)). Whereas the nature of this binding appears to be similar to that of Zn^2+^-bound MamM CTD, neither Cd^2+^- nor Ni^2+^-bound crystal structure models would suggest such a metal-dependent conformational change. Consequently, MMM inter-spin distance predictions are 4.14 and 4.11 nm for Cd^2+^- and Ni^2+^-bound structures, respectively (Fig. 5, *B* and *C*). Because the occupancy for each metal-binding site in the Cd^2+^-bound solved crystal structure is rather low (0.5), and considering the ITC data which indicate that only one Cd^2+^ ion is bound per dimer, and the observed change in tryptophan fluorescence, this would suggest that in solution only one Cd^2+^ binds at the CS. This is in contrast to Zn^2+^ binding because it appears that only metal binding at the CS is required to induce the conformational change. This is possible because of the larger size of the Cd^2+^ ion which, once bound to the CS residues of just one monomer, is in close enough proximity to attract the CS binding residues of the second monomer, forcing the observed conformational change. The observed conformational change with Ni^2+^ might be rationalized by nonspecific Ni^2+^-binding events, as suggested by the metal-ligand variations within the solved structure. Nonetheless, the shallower modulation depth (Fig. 6*E*) may indicate that a smaller fraction of nitroxide spins are actually dipolar coupled and thus interacting. ITC results indicate binding of three Ni^2+^ ions per dimeric protein with mild affinity compared with the other metals, and the observation of tryptophan fluorescence quenching in the presence of Ni^2+^ would also imply Ni^2+^ binding either at or close to the CS. Taken together, and considering that the nickel ion's radius is comparable with that of a zinc ion, we propose that Ni^2+^ binds both to the CS (one ion) and the PSs (two ions), with binding to the latter being associated with a conformational change similar to that observed for Zn^2 +^.

The room-temperature cw-EPR experiments of C267S_SL_ incubated with an excess of Cu^2+^, which is itself paramagnetic (forming C267S_SL_-Cu^2+^), suggest a partial quenching of the EPR signal as compared with both the Zn^2+^ bound and apo states (Fig. 6*A*). The Cu^2+^ ions are EPR active (S = 1/2), giving EPR signals that spectrally overlap with that of the spin label; thus the excess chosen for PELDOR measurements was equal to the number of proposed binding sites to avoid any exogenous, spurious Cu^2+^ EPR signals arising from nonspecifically bound copper. MMM analysis of the Cu^2+^-bound structure (Fig. 5*D*) predicts an inter-spin label distance of 3.23 nm, whereas the resultant distance distribution of the experimental PELDOR trace reveals a bimodal distribution with two mean distances of 2.4 and 3.24 nm (Fig. 6*F*). At first glance this could imply two different inter-spin label (SL-SL) distances; however, because the Cu^2+^ species overlaps with the spin label in the EPR spectrum, this PELDOR trace may not only comprise SL-SL distances but in addition contributions from SL-Cu^2+^ and Cu^2+^-Cu^2+^ distances. The 3.24-nm distance agrees well with both the predicted MMM SL-SL distance and other metal-bound PELDOR results, and hence we assign this to the SL-SL distance, again indicative of a single closed V-shaped conformation. The second observed distance at 2.4 nm could correspond to either a Cu^2+^-SL or Cu^2+^-Cu^2+^ distance. Considering the fluorescence data, which indicates binding of Cu^2+^ in the CS, and previous PELDOR measurements in which conformational change was shown to be associated with metal binding to the PSs (21), we propose that binding to the CS is silent in terms of heat change and that the Cu^2+^-bound crystal structure represents well both the conformation in solution and the copper binding sites.

Current experimental evidence for Fe^2+^ binding to MamM CTD, both via biophysical and structural methods, is very limited (5). This is due largely to the difficulty associated with preparation of bound Fe^2+^ samples; when working at basic pH, Fe^2+^ is readily oxidized to Fe^3+^, which notoriously forms insoluble complexes. Nevertheless, it proved possible to prepare an Fe^2+^-incubated sample using argon gas to largely eliminate any oxygen present with C267S_SL_ and an excess of Fe^2+^ (×5 excess, forming C267S_SL_-Fe^2+^). Although there was slight indication of Fe^3+^ precipitation, PELDOR yielded results clearly different from the apo state: nonbaseline-corrected, time-domain data are shown to indicate these differences more clearly, and a single distance distribution with a mean distance of 3.3 nm is obtained (Fig. 6*G*). Despite the low modulation depth, probably due to very low levels of Fe^2+^ binding, the difference to the apo state is quite apparent, providing a good indication of a more rigid structure although the distance distribution is slightly broader than for Zn^2+^/Ni^2+^/Cu^2+^/Cd^2+^. Interestingly, the sample became slightly yellow upon incubation with the metal salt, which could not be reproduced upon incubation of the buffer alone with the same concentration of salt. It is therefore likely that the protein also catalyzes the iron oxidation process, resulting in the formation of unbound (or nonspecifically bound) yellow Fe^3+^ complexes. More importantly, this is the first high-resolution structural observation of Fe^2+^-binding to MamM CTD which corroborates our earlier findings of iron binding and the associated conformational change of MamM CTD upon binding (5). These results indicate that studies with other metals (including Zn^2+^, for which substantial work has been done previously) do reflect biologically relevant states of the protein, because all the metal-bound proteins appear to adopt a similar conformation in solution, which is most likely similar to the crystal Cu^2+^-bound conformation.

In contrast, the addition of paramagnetic Mn^2+^ (×5 excess) to C267S_SL_ does not result in a change in the dipolar evolution time trace seen when other divalent metals are added (Fig. 6*B*). There are no periodic oscillations arising from dipolar coupling between the two nitroxide moieties but simply only a slightly modified decay function modulated by the presence of deuterated glycerol (which are not present when protonated glycerol is used, data not shown). Thus, PELDOR spectroscopy, taken together with the tryptophan fluorescence and ITC data, provide clear evidence of the inability of MamM CTD to bind Mn^2+^.

Previous analysis of metal-binding sites of all PDB structures revealed that the typical amino acid composition in Mn^2+^ binding sites is similar to those that bind Fe^2+^, Cd^2+^, Zn^2+^, and Ni^2+^ (7), and MamM's cation binding sites are composed of histidine residues and aspartic or glutamic acids; however, MamM CTD can bind these cations but still discriminates against Mn^2+^. This phenomenon cannot be rationalized by comparison of other properties important for metal binding such as dominant binding geometry (tetrahedral, octahedral, or trigonal plane), metal size, solvation sphere, or electrostatic charges. Attempts to actively modify the MamM CTD metal-binding sites to allow Mn^2+^ binding *in vitro* failed to alter the CTD discrimination abilities (28). Overall, the common binding capabilities of Mn^2+^ throughout biology indicate that the observed discrimination by MamM CTD must be more complex and may include quantum effects that cannot be calculated for this system. The inability of MamM CTD to bind Mn^2+^, in contrast to the other cations, is further indication of an evolutionary pressure that discriminates against Mn^2+^.

**Figure 7:**
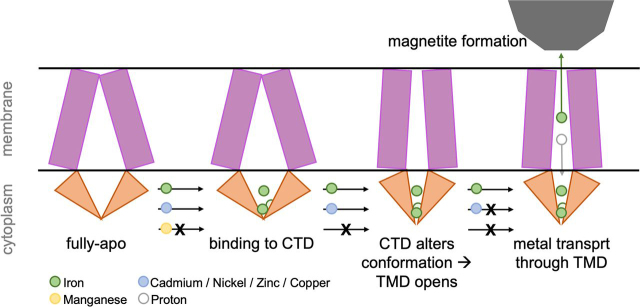
Metal-binding mechanism to MamM CTD. MamM CTD can bind divalent iron (*green sphere*), cadmium, zinc, nickel, and copper (*blue sphere*), but not manganese (*yellow sphere*). Metal binding to the CTD leads to its tighter packing, which in turn facilitates the conformational change of the transmembrane domain. However, only iron can be transported through this domain, in exchange for protons, which allows the synthesis of pure magnetic particles.

Our results ultimately raise the question of what benefits there are for MamM CTD as a result of its discrimination against Mn^2+^. Mn^2+^ has a greater affinity to magnetite, both when abiotically synthesized and in magnetotactic bacteria, as compared with other transition metal cations (29). It was shown that for iron oxides formation *in vitro*, the presence of manganese leads to oxidation of Fe^2+^ to Fe^3+^ by manganese oxides (30); thus it may change the proper magnetite formation in the magnetosome by changing the Fe^2+^:Fe^3+^ balance. Furthermore, uncultivated magnetotactic bacteria, which are exposed to very high concentrations of manganese, can incorporate it into the iron-based magnetic particles, yet to a limited amount (31). Based on our results and these observations, we propose that to avoid overflow of manganese in the magnetosomes, leading to sequential incorporation of it into magnetite or altering magnetite formation by affecting the oxidation/reduction of iron, evolutionary demands have driven MTB to develop a CDF CTD-related mechanism for discrimination against manganese. Such discrimination will prevent both Mn^2+^ ion transport and any initial activation of the transporter by not facilitating any binding of Mn^2+^ to the CTD (Fig. 7). Whereas all the other tested metals were shown to bind to the CTD, leading to a tighter conformation similar to that of the iron-bound CTD, only rarely are contaminated magnetite particles formed in MTB *in vivo*. Our results would thus indicate that any discrimination against other metals should occur at the transport site level, which is unique compared with other CDF proteins (contains a rare tyrosine residue) (7, 18) (Fig. 7).

### Concluding remarks

The participation of CDF protein CTDs in metal selectivity was previously studied at the cellular level. Here, for the first time, structural characterization of a CDF protein CTD in the presence of various metals reveals the direct effect of different metals on the CTD, with each metal exhibiting distinct binding parameters, sites, and conformation. This study uniquely includes high-resolution structural data both in the crystalline form and in solution, which are complementary to one another and in combination exhibit the differences between the binding modes to the native metal and other metals. Furthermore, these results present subtle differences in the degree-of-closure between the apo and bound CTDs among different CDF proteins, suggesting that it not only depends on the protein studied but, importantly, also on the nature of the metal. Based on these distinct binding modes, we propose that the CDF CTD is the first recognition site able to alter or stop the transport of the unwanted metals prior to them reaching the transport site, which provides a second recognition site. This could be because of the slightly different CTD closed conformations induced by differences in the metal size, metal coordination geometry, or binding of the flexible C-terminal tail. Such an additional ability of the CTD to discriminate would also mean that the CDF is not simply limited by a single site, making it hard to discriminate against all the different cations that exist in the cytoplasm because of their relative similarity. One surprising piece of evidence for such varying levels of discrimination is found in the MamM system where manganese is fully discriminated against by the CTD alone, which may reflect the specific evolutionary pressure to specifically transport iron.

## Experimental procedures

### Expression and purification

*mamM* CTD gene (UniProt Q6NE57 residues 215–318) was previously cloned into pET28a(+) vector (Novagen, Merck Biosciences, Germany) (32). MamM CTD was expressed and purified as previously described for MamM CTD M250L mutant (20, 32). The C267S mutation for PELDOR studies was applied to the pET28a-MamM CTD vector, expressed, and purified as previously described (21). For all experiments, protein concentration was determined by measuring protein absorption at 280 nm.

### Crystallization and structure determination

20 mg ml^−1^ purified MamM CTD in buffer containing 10 mm Tris, pH 8.0, 150 mm NaCl, 5 mm β-mercaptoethanol, and either 3.375 mm CuSO_4_ (Cu^2+^-bound structure), 3.375 mm CdCl_2_ (Cd^2+^-bound structure), or 3.375 mm NiCl_2_ (Ni^2+^-bound structure) was crystallized using the vapor diffusion method at 293 K (0.3 µl of protein with 0.3 µl of reservoir solution for all protein-metal pairs). Crystals were harvested with or without treatment of cryo-agent and flash-frozen in liquid nitrogen. Data collection was performed on a single crystal at 100 K. All cryo conditions, data collection details, data reduction, and scaling, phasing, and refinement details are given in Tables S1–S3 (37, 38, 39, 40, 41, 42, 43).

### Least-squares overlaps

All CDFs' CTD structures were overlapped and RMSDs were calculated using the iterative magic fit tool or the domain fragment alternate fit tool of Swiss-PdbViewer 4.1.0 (33). MamM CTD structures and overlapped structures' figures were prepared using UCSF Chimera package, version 1.12 (34) or PyMOL, version 2.0.6 (PyMOL Molecular Graphics System, Schrödinger).

### Fluorescence spectrometry

Changes in tryptophan intrinsic fluorescence were recorded using Fluorolog®-3 (HORIBA, Edison, NJ, USA) equipped with a quartz cell with a 1-cm optical path at ambient temperature. Samples of 1 ml of MamM CTD at 5 µm concentration in buffer A (containing 10 mm Tris, pH 8.0, and 150 mm NaCl) were titrated using 2.5 mm metal solution (CdCl_2_, NiCl_2_, MnCl_2_, and CuSO_4_) in the same buffer to reach different concentrations. Samples were measured at λ_ex_ 297 nm, and the emission spectrum for each metal concentration was recorded at 310–450 nm. For each metal, the titration was replicated three times, and each spectrum was fitted to Extreme function by OriginPro (R-Square > 0.98) (OriginLab Corporation). The maximum wavelength (wavelength at maximum intensity) and the intensity at that wavelength (maximum intensity) were averaged for each metal concentration. Errors are reported as the standard deviation.

### Isothermal titration calorimetry

ITC measurements were performed in a low-volume Nano ITC calorimeter (TA Instruments) at 298 K. Proteins and metals (CdCl_2_, NiCl_2_, MnCl_2_, and CuSO_4_) were prepared in buffer A. MamM CTD was diluted to 50 µm concentration and was titrated with 1.4 mm (NiCl_2_, MnCl_2_, and CuSO_4_) or 1 mm (CdCl_2_) metal solutions. The protein samples were injected into the instrument cell (170 µl), and 20 aliquots of 2.5 µl of the suitable metal solution were titrated into the cell every 350 s. For each protein, three independent titrations were measured. As a control, each metal solution was titrated into buffer A under the same experimental conditions, and all measurements were compared with a DDW-containing reference cell. Data were analyzed using TA NanoAnalyze Data Analysis software, version 3.7.5 (TA Instruments). The data of each measurement were fitted to an independent model combined with a blank constant model, and the given thermodynamics values are the average of the three different titrations for each protein. Errors are reported as standard deviation.

### Site-directed spin labeling

For site-directed spin labeling, protein solutions in buffer A were degassed under argon and treated with degassed DTT (1 mm) for 4 h at 4 °C with gentle agitation. Subsequently, protein samples were washed once using Zeba^TM^ Spin desalting columns (7 kDa molecular weight cutoff, 2 ml) (Thermo Fisher Scientific) equilibrated with degassed buffer A. Protein was then labeled overnight at 4 °C with (1-oxyl-2,2,5,5-tetramethylpyrrolidin-3-yl)methylthiosulfonate spin label (MTSL, 20× excess) (Toronto Research Chemicals) with gentle agitation. To remove excess unbound MTSL, the samples were washed twice using Zeba^TM^ Spin desalting columns equilibrated with degassed buffer A.

### Continuous-wave X-band EPR spectroscopy

For metal-bound samples, freshly spin-labeled C267S MamM CTD in buffer A at ∼70 µm was used. Stock solutions of metal salts (ZnSO_4_.5H_2_O and CuCl_2_.2H_2_O) were prepared in H_2_O to a final concentration of 100 mm. Aliquots (15 µl) of spin-labeled protein were incubated with a 3-fold excess of each metal salt for 40 min on ice. Aliquots of ∼10 µl were transferred to 0.8-mm (outer diameter) capillary tubes for measurement.

The ambient temperature setup for X-band cw-EPR consisted of a Bruker E500 eleXsys spectrometer fitted with an ER 4123D (dielectric RT cw-EPR) resonator. The following measuring parameters were used for data acquisition: a microwave frequency of 9.758 GHz, a modulation frequency of 100 KHz, a modulation amplitude of 1 gauss, and a microwave power of 0.2 milliwatt.

### Pulsed EPR spectroscopy

Spin-labeled C267S MamM CTD samples were lyophilized and subsequently dissolved in D_2_O to a final concentration of 250 µm (Cd^2+^- and Fe^2+^-bound samples) or 500 µm (Mn^2+^-, Ni^2+^-, and Cu^2+^-bound samples). All EPR samples were prepared with 30% Glycerol-d_8_ (Sigma-Aldrich). Stock solutions of metal salts (CdCl_2_, MnCl_2_.7H_2_O, NiCl_2_, CuSO_4_.5H_2_O, and Fe(NH_4_)_2_(SO_4_)_2_.6H_2_O) were prepared to a final concentration of 100 mm in D_2_O. Spin-labeled samples were incubated with a 5-fold (Cd^2+^-, Mn^2+^-, Ni^2+^-, and Fe^2+^-bound samples) or 3-fold (Cu^2+^-bound) excess according to the dimeric protein concentration. Incubation was carried out at room temperature for 1 h, before the addition of glycerol-d_8_ to a final concentration of ∼30%. 80–100-µl samples were transferred to EPR quartz tubes (Wilmad SQ-707; Wilmad-LabGlass) and flash-frozen in liquid nitrogen. For the Fe^2^-bound sample, each step was carried out under a steady stream of argon to eliminate oxygen during sample preparation. X-band pulsed EPR spectra were recorded on a Bruker E580 spectrometer (Bruker, Rheinstetten, Germany) using a Bruker MD5-W1 EPR probehead equipped with a self-modified cryogen-free cryostat (Advanced Research Systems, Macungie, PA, USA). The microwave pulses were amplified using a 1-kW TWT amplifier (Applied Systems Engineering, Fort Worth, TX, USA). All EPR experiments were carried out at 50 K. The field-swept spectrum was obtained by integrating the Hahn echo signal as a function of the magnetic field after a two-pulse sequence (10 ns, τ(120 ns), 20 ns). For PELDOR experiments a four-pulse sequence was applied. Observer pulses (π/2 and π) were set to 20 ns, with a pump pulse of 16 ns. The frequency offset Δν (ν_obs_ − ν_pump_) was set to between 70–80 MHz for SL-SL measurements. The observer pulse separation τ_1_ was 120 ns in all cases apart from with Ni^2+^, where τ_1_ was 350 ns, whereas τ_2_ varied between 1300–2200 ns, and echo signals were integrated using a video amplifier bandwidth of 20 MHz. The pump pulse was stepped out in 12-ns intervals to a total number of points that depended upon the τ_1_ and τ_2_ used. Nuclear modulation effects were suppressed using a systematic variation of the inter-pulse delay time τ_1_ and an appropriate phase cycling routine.

PELDOR data processing was performed using both Tikhonov regularization and DEERNet (35) within the Matlab-based DEER Analysis (36) toolbox. The recently developed DEERNet method uses artificial neural networks for prediction of an inter-spin label distance distribution and gives a measure of uncertainty of the resulting distance distributions represented by 95% confidence bounds, which are indicated as the *gray bands* in Fig. 6.

## Data availability

All structural data are available in RCSB Protein Data Bank under the accession numbers 6GP6, 6GMT, and 6GMV. All other data are contained within this article and in the supporting information.
