# Supplementary material for: The cation diffusion facilitator protein MamM's cytoplasmic domain exhibits metal-type dependent binding modes and discriminates against Mn2+
Source: J Biol Chem. 2021 Jan 13;295(49):16614–29. doi: 10.1074/jbc.RA120.014145 (PMC7864060; doi:10.1074/jbc.RA120.014145)
Supplement: Supplementary file 1 [file mmc1.pdf]

**Supporting Information for:**

# The cation diffusion facilitator protein MamM's cytoplasmic domain exhibits metal-type dependent binding modes and discriminates against $Mn^{2+}$

Shiran Barber-Zucker[1,2,3,¥], Jenny Hall[4,¥], Afonso Froes[4], Sofiya Kolusheva[3], Fraser MacMillan[4,*] and Raz Zarivach[1,2,3,*]

[1] Department of Life Sciences, Ben-Gurion University of the Negev, Beer Sheva 8410501, Israel

[2] The National Institute for Biotechnology in the Negev, Ben-Gurion University of the Negev, Beer Sheva 8410501, Israel

[3] Ilse Katz Institute for Nanoscale Science and Technology, Ben-Gurion University of the Negev, Beer Sheva 8410501, Israel

[4] Henry Wellcome Unit for Biological EPR, School of Chemistry, University of East Anglia, Norwich Research Park, Norwich NR4 7TJ, United Kingdom

**List of the material included:**

Table S1: Crystallization of MamM CTD with different metals.

Table S2: Data collection and refinement statistics of MamM CTD with different metals.

Table S3: Crystallographic software used for structure solution of MamM CTD with different metals.

Table S4: Dihedral angles and distances between selected residues in all CDFs' CTD structures.

Table S1: Crystallization of MamM CTD with different metals.

| Protein name | **MamM CTD $Cu^{2+}$-bound** | **MamM CTD $Cd^{2+}$-bound** | **MamM CTD $Ni^{2+}$-bound** |
|---|---|---|---|
| PDB ID | 6GP6 | 6GMT | 6GMV |
| Crystallization conditions | 0.2 M NaCl, 0.1 M TRIS pH 8.7, 25% PEG 3350 | 0.2 M $Li_2SO_4$, 0.1 M BIS-TRIS pH 5.7, 25% PEG 3350 | 0.2 M $(NH_4)_2SO_4$, 0.1 M BIS-TRIS pH 5.5, 25% PEG 3350 |
| Cryo protectant | 50% PEG 3350 | - | - |
| Protein concentration (mg $mL^{-1}$) | 10 | 10 | 10 |
| Crystallization type | Vapor diffusion (sitting drop) | | |
| Data collection | ESRF – ID30A3 | ESRF – ID30A3 | ESRF – ID23-1 |
| Detector | Eiger X 4M | Eiger X 4M | Pilatus 6M |

Table S2: Data collection and refinement statistics of MamM CTD with different metals.

| Protein name | **MamM CTD $Cu^{2+}$-bound [a]** | **MamM CTD $Cd^{2+}$-bound** | **MamM CTD $Ni^{2+}$-bound** |
|---|---|---|---|
| PDB ID | 6GP6 | 6GMT | 6GMV |
| Data collection | ESRF – ID30A3 | ESRF – ID30A3 | ESRF – ID23-1 |
| Space group | $P\ 2\ 2_1\ 2_1$ | $C\ 2\ 2\ 2_1$ | $C\ 2\ 2\ 2_1$ |
| ***Cell dimensions*** | | | |
| a, b, c (Å) | 28.93, 73.75, 89.41 | 36.53, 94.25, 53.32 | 37.34, 94.48, 53.69 |
| α, β, γ (°) | 90, 90, 90 | 90, 90, 90 | 90, 90, 90 |
| Resolution (Å) | 2.14-44.71 (2.14-2.20) [b] | 1.59-47.12 (1.59-1.61) | 1.59-47.24 (1.59-1.62) |
| Rsym or Rmerge | 0.106 (1.863) | 0.027 (1.046) | 0.059 (2.376) |
| I/σI | 8.5 (1.0) | 20.6 (1.2) | 24.3 (1.4) |
| $CC_{1/2}$ | 0.996 (0.436) | 1.000 (0.445) | 1.000 (0.769) |
| Completeness (%) | 99.6 (96.9) | 98.2 (97.9) | 99.9 (97.4) |
| Redundancy | 5.8 (5.8) | 3.9 (4.0) | 19.4 (19.1) |
| Wavelength (Å) | 0.96771 | 0.96771 | 0.977999 |
| No. unique reflections | 11127 (864) | 12566 (597) | 13052 (616) |
| ***Refinement*** | | | |
| Resolution (Å) | 2.15-44.71 (2.15-2.46) | 1.59-47.12 (1.59-1.63) | 1.59-47.24 (1.59-1.64) |
| Rwork/Rfree | 22.01/27.51 (25.6/34.5) | 18.68/23.93 (40.0/39.9) | 18.82/22.18 (33.9/38.5) |
| *No. atoms* | | | |
| Protein | A-796, B-714 | 637 | 664 |
| Ligand/ion | 7 | 16 | 15 |
| Water | 21 | 53 | 64 |
| *B-factors* | | | |
| Protein | A-40.12, B-47.18 | 34.95 | 35.94 |
| Ligand/ion | $Cu^{2+}$-76.95, βME-50.81 | $Cd^{2+}$-43.55, $SO_4$-38.82, βME-60.39 | $Ni^{2+}$-68.24, $SO_4$-38.59, βME-73.73 |
| Water | 41.44 | 42.77 | 44.10 |
| *RMSD* | | | |

| | | | |
|---|---|---|---|
| Bond lengths (Å) | 0.009 | 0.028 | 0.031 |
| Bond angles (°) | 1.076 | 2.620 | 2.595 |
| Ramachandran statistics [c] | P: 181 (97.31%), A: 2 (1.08%), O: 3 (1.61%) | P: 76 (98.70%), A: 1 (1.30%), O: 0 (0%) | P: 71 (98.61%), A: 1 (1.39%), O: 0 (0%) |
| Missing residues | A: 211, 315-318 B: 302-318 | 211-212, 293-318 | 211, 293-318 |

Values in parentheses are for the highest resolution shell.

One crystal was used per dataset.

Datasets were collected at 100K.

[a] Collection statistics are given after the Aimless scaling; data were further processed with UCLA-DOE-LAB Diffraction Anisotropy Server (http://services.int.mbi.ucla.edu/anisoscale/).

[b] Best resolution is given for b axis (best resolution is 2.3 Å and 2.6 Å for a and c axes, respectively, after anisotropic data reduction).

[c] P- Preferred region, A- Allowed region, and O- outliers.

Table S3: Crystallographic software used for structure solution of MamM CTD with different metals.

| Protein name | **MamM CTD $Cu^{2+}$-bound** [a] | **MamM CTD $Cd^{2+}$-bound** | **MamM CTD $Ni^{2+}$-bound** |
|---|---|---|---|
| PDB ID | 6GP6 | 6GMT | 6GMV |
| Data reduction | XDS (37) | | |
| Data scaling | Aimless (38) [a] | Aimless | Aimless |
| Structure solution method | Molecular replacement – using MamM CTD wildtype structure (PDB ID: 3W5X) | | |
| Phasing | Phaser MR (39) | | |
| Refinement | Phenix (40) | Refmac5 (41) | Refmac5 |

Manual refinement was performed using Coot version 0.8.9 (42).

Aimless, Phaser MR and Refmac5 were used through the CCP4i package (43).

[a] After Aimless scaling, data were further processed with UCLA-DOE-LAB Diffraction Anisotropy Server.

Table S4: Dihedral angles and distances between selected residues in all CDFs' CTD structures.

| | Distance (Å) | | | Dihedral angle (°) | |
|---|---|---|---|---|---|
| Protein form | R240-R240 | G276-G276 | V242-V242 | R240-P256-P256-R240 | V242-V260-V260-V242 |
| MamM apo (3W5X) | 25.78 | 45.40 | 30.03 | 48.759 | -47.116 |
| MamM apo (3W5Y) | 22.86 | 44.31 | 27.34 | 43.240 | -41.640 |
| MamM $Cu^{2+}$-bound (6GP6) | 21.47 | 39.57 | 19.39 | 28.533 | -34.863 |
| MamM $Cd^{2+}$-bound (6GMT) | 26.18 | 45.11 | 29.23 | 48.110 | -48.105 |
| MamM $Ni^{2+}$-bound (6GMV) | 26.34 | 45.51 | 30.01 | 50.176 | -47.742 |
| | R234-R234 | Q270-Q270 | A236-A236 | R234-G250-G250-R234 | A236-V254-V254-A236 |
| CzrB apo (3BYP) | 28.13 | 45.54 | 30.96 | 51.849 | -51.592 |
| CzrB bound (3BYR) | 12.22 | 37.60 | 13.16 | 21.354 | -14.796 |
| | R237-R237 | R273-R273 | S239-S239 | R237-D253-D253-R237 | S239-L257-L257-S239 |
| EcYiiP bound (3H90) | 14.26 | 36.86 | 13.89 | 23.880 | -16.838 |
| | R239-R239 | A275-A275 | A241-A241 | R239-G255-G255-R239 | A241-L259-L259-A241 |
| SoYiiP bound (5VRF) | 15.28 | 39.11 | 15.19 | 23.801 | -17.963 |
| | R238-R238 | A274-A274 | V240-V240 | R238-A254-A254-R238 | V240-V258-V258-V240 |
| MamB apo (5HO5) | 19.44 | 39.79 | 17.10 | 28.135 | -30.799 |
| MamB bound (5HO1) | 19.10 | 38.93 | 18.25 | 26.612 | -32.822 |

All distances and dihedral angles were measured between the residues' $C_\alpha$ from both monomers, using UCSF Chimera package, version 1.12 (34).

All the residues in each column are found in the same location in all the proteins.
